# Supplementary material for: AI-driven 3D CT imaging prediction model for improving preoperative detection of visceral pleural invasion in early-stage lung cancer
Source: PLoS One. 2025 Oct 17;20(10):e0332956. doi: 10.1371/journal.pone.0332956 (PMC12533904; doi:10.1371/journal.pone.0332956)
Supplement: S1 File — (DOCX) [file pone.0332956.s001.docx]

**Supporting information**

**Supplementary Methods**

**Patient cohort**

This study was a retrospective, single-center, observational study. Detailed inclusion and exclusion criteria were provided here to add to the Manuscript. We also described the details of the preoperative staging in this Method.

A total of 556 patients with clinical stage 0 to I NSCLC who received complete surgical resection at Tokyo Medical University Hospital between January 2011 and December 2018 were included in the study. We focused particularly on tumors located near the pleural surface as identified on preoperative thin-section CT scan for the purpose of predicting organ pleural invasion (VPI) in lung cancer. We excluded patients with tumors larger than 4 cm in size, as these are classified as stage II or higher according to the 8th TNM classification and VPI does not alter their staging. In addition, previous studies have shown that in tumors >3 cm, VPI has limited impact on prognosis and nodal metastasis (1-3). Patients with tumors not adjacent to the pleural surface on preoperative CT images or with preoperative CT scans that could not be analyzed by the AI software beta version of the Synapse Vincent system (Fujifilm Corporation, Tokyo, Japan) were also excluded. Patients who had received preoperative chemotherapy and/or radiotherapy were excluded from the study.

The preoperative stage was determined by HRCT, 18F-fluorodeoxyglucose (FDG) positron emission tomography/computed tomography (PET/CT), or endobronchial ultrasound-guided transbronchial needle aspiration (EBUS-TBNA) followed by histological analysis. Regarding the clinical node assessment, the absence of lymph node metastasis was defined as enlarged lymph nodes less than 10 mm in the short axis on CT images or negative FDG uptake by the lymph nodes on PET/CT images. Invasive examinations for mediastinal lymph node staging, such as EBUS-TBNA, were performed preoperatively when the clinical node criteria showed radiologically positive metastasis. Clinical staging was performed based on the TNM classification (8th edition) (4). Our institutional review board approved the study protocol for data collection and analyses and waived the need for informed consent from each patient (study approval no: SH3951).

**Radiological evaluation**

HRCT images of the whole lung were acquired using the settings previously described (5). Detailed information was provided here to add to the Manuscript.

Briefly, HRCT images with a 1.25-mm thickness were obtained of the entire lung. The whole tumor size and solid tumor size were preoperatively measured on the HRCT images. The solid tumor size was defined as the maximum dimension of the solid component of the lung window, excluding Ground-glass nodules (GGNs). The CTR (%) was defined as the maximum dimension of consolidation in the lung window setting divided by the maximum dimension of the tumor in the lung window setting.

**Artificial intelligence analysis with Synapse Vincent**

We utilized the AI software Beta Version (AI software; Fujifilm Corporation) of Synapse Vincent system (Fujifilm Corporation, Tokyo, Japan) for analysis. Detailed information was provided here to add to the Manuscript.

This segmentation algorithm is based on a 3D convolutional neural network using a modified U-net architecture. This network consisted of 17 convolutional layers. The latest version of the development software has enhanced capabilities, automating the detection and extraction of pulmonary nodules across the entire lung field. With the activation of the computer-aided detection (CAD) system, it can identify and calculate the volumes of GGNs and solid lesions, along with their respective proportions. The system automatically calculates 17 three-dimensional radiological parameters, including the maximum diameter of the tumor, the solid component, and the CT values.

Additionally, this AI software beta version in the Synapse Vincent system facilitates the characterization of pulmonary nodules by assessing 22 radiological features, quantifying each with a confidence score ranging from 0 to 1. The 22 radiological features were based on the labeling of 5,118 tumors. The datasets of the development process were divided into training, validation, and test sets. The trained model yielded a mean area under the curve (AUC) score of 0.93 for all features in the test dataset (6, 7). This AI lung nodule analysis model uses a convolutional neural network based on VGG-16 and consists of 12 convolution layers, with four layers removed from the output side of the VGG-16. To extract 3D imaging features, 3D convolution was used for all convolution layers.

A detailed description and figure of the 22 AI-derived radiological features used in the analysis is provided below (Fig S5). These features were automatically extracted from CT images using the AI algorithm. Following the methodology described in reference (7), representative image examples with high AI confidence scores were selected for each of the 22 features.

**Irregular shape**: Tumors with an asymmetrical or uneven contour.

**Round shape**: Tumors with a generally circular or oval outline.

**Smooth shape**: Tumors with a uniformly flat and regular surface.

**Clear boundary**: The tumor surface is distinctly visible and can be clearly differentiated from the surrounding lung parenchyma.

**Serrated edge**: Tumor edge exhibits a saw-tooth-like appearance.

**Spiculation**: Thin lines extending from the edge of the tumor into the surrounding lung tissues.

**Lobulated edge**: Tumor border appears multilobulated.

**Polygon edge**: Tumor outline displays multiple straight segments forming polygonal shapes.

**Bronchus translucency**: Presence of bronchial air passages visible within the tumor.

**Bronchial convergence**: Bronchi appear to converge toward the tumor mass.

**Bronchial compression**: Narrowing of bronchi due to tumor pressure.

**Pleural indentation**: Local retraction of the pleura toward the tumor.

**Pleural contaction**: Tumor contact to the pleural surfaces.

**Pleural recess**: Tumor extension into the pleural recesses.

**Pleural hypertrophy**: Thickening of pleura adjacent to the tumor.

**Solid**: Tumor appears completely opaque without air or fluid components.

**Part-solid**: Tumor contains both solid and ground-glass components.

**Ground-glass opacity (GGO)**: Tumor area with hazy increased attenuation that does not obscure underlying structures.

**Cavity**: Air-filled space within the tumor mass.

**Calcification**: Presence of high-density foci within the tumor indicating calcified material.

**Fat component**: Tumor shows areas with fat-density attenuation.

**Pathological evaluation**

All resected specimens were formalin-fixed and stained with hematoxylin and eosin using routine procedures. Detailed information was provided here to add to the Manuscript.

Experienced pathologists (J.M. and T.N.) reviewed the surgical specimens. Pathological staging was performed using the TNM classification (7th edition) (8). Histopathological analyses were performed according to the World Health Organization criteria (4th edition) (9). Elastica van Gieson (EVG) and D2-40 staining routinely evaluate histological structures and tumor invasion. VPI was defined as tumor invasion beyond the elastic layer of the pleura. The BVI and lymphatic permeation were determined by identifying conspicuous clusters of intravascular cancer cells surrounded by blood and lymphatic vessels.

**Supplementary References**

1. Shimizu K, Yoshida J, Nagai K, Nishimura M, Yokose T, Ishii G, et al. Visceral pleural invasion classification in non-small cell lung cancer: a proposal on the basis of outcome assessment. J Thorac Cardiovasc Surg. 2004;127(6):1574-8.

2. Yoshida J, Nagai K, Asamura H, Goya T, Koshiishi Y, Sohara Y, et al. Visceral pleura invasion impact on non-small cell lung cancer patient survival: its implications for the forthcoming TNM staging based on a large-scale nation-wide database. J Thorac Oncol. 2009;4(8):959-63.

3. Kudo Y, Saji H, Shimada Y, Nomura M, Matsubayashi J, Nagao T, et al. Impact of visceral pleural invasion on the survival of patients with non-small cell lung cancer. Lung cancer. 2012;78(2):153-60.

4. Goldstraw P, Chansky K, Crowley J, Rami-Porta R, Asamura H, Eberhardt WE, et al. The IASLC Lung Cancer Staging Project: Proposals for Revision of the TNM Stage Groupings in the Forthcoming (Eighth) Edition of the TNM Classification for Lung Cancer. J Thorac Oncol. 2016;11(1):39-51.

5. Kudo Y, Matsubayashi J, Saji H, Akata S, Shimada Y, Kato Y, et al. Association between high-resolution computed tomography findings and the IASLC/ATS/ERS classification of small lung adenocarcinomas in Japanese patients. Lung Cancer. 2015;90(1):47-54.

6. Momoki Y, Ichinose A, Shigeto Y, Honda U, Nakamura K, Matsumoto Y. Characterization of pulmonary nodules in computed tomography images based on pseudo-labeling using radiology reports. IEEE Transactions on Circuits and Systems for Video Technology. 2021;32(5):2582-91.

7. Momoki Y, Ichinose A, Nakamura K, Iwano S, Kamiya S, Yamada K, et al. Development of automatic generation system for lung nodule finding descriptions. Plos one. 2024;19(3):e0300325.

8. Sobin LH, Gospodarowicz MK, Wittekind C. TNM classification of malignant tumours: John Wiley & Sons; 2011.

9. Travis WD, Brambilla E, Burke A, Nicholson AG. WHO Classification of Tumours of the Lung, Pleura, Thymus and Heart: International Agency for Research on Cancer; 2015.
