# Supplementary figures and images for: AI-driven 3D CT imaging prediction model for improving preoperative detection of visceral pleural invasion in early-stage lung cancer
Source: PLoS One. 2025 Oct 17;20(10):e0332956. doi: 10.1371/journal.pone.0332956 (PMC12533904; doi:10.1371/journal.pone.0332956)

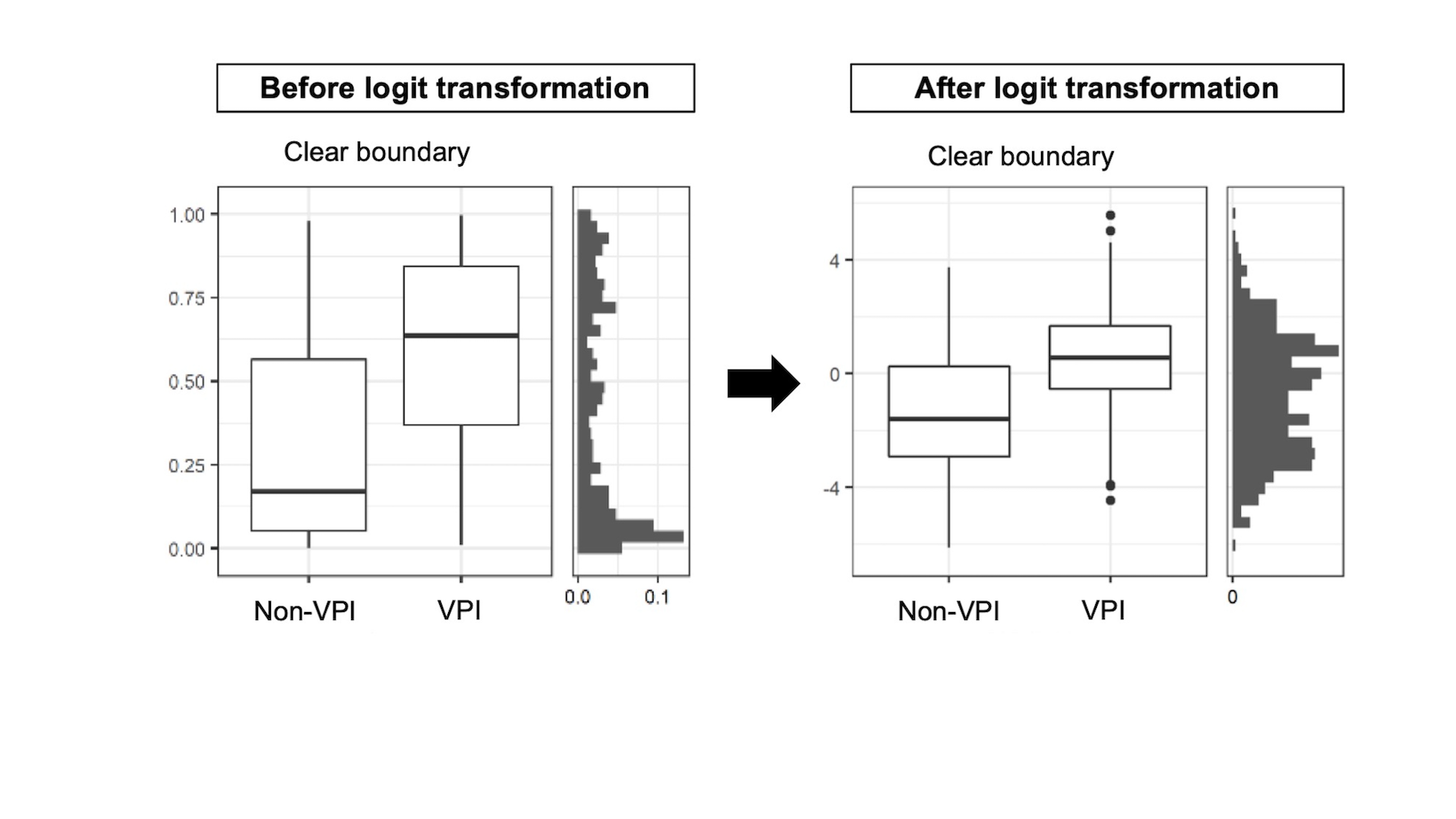

Supplement: S1 Fig — This is the Fig S1 legend. An example of the distribution of AI confidence scores before and after the logit transformation is shown. Before the transformation, the distribution is skewed towards 0 or 1, and it is assumed that there is a nonlinear relationship with the target variable due to ceiling or floor effects. After the logit transformation, the variable follows a more spread-out distribution. (TIFF) [file pone.0332956.s002.tiff]

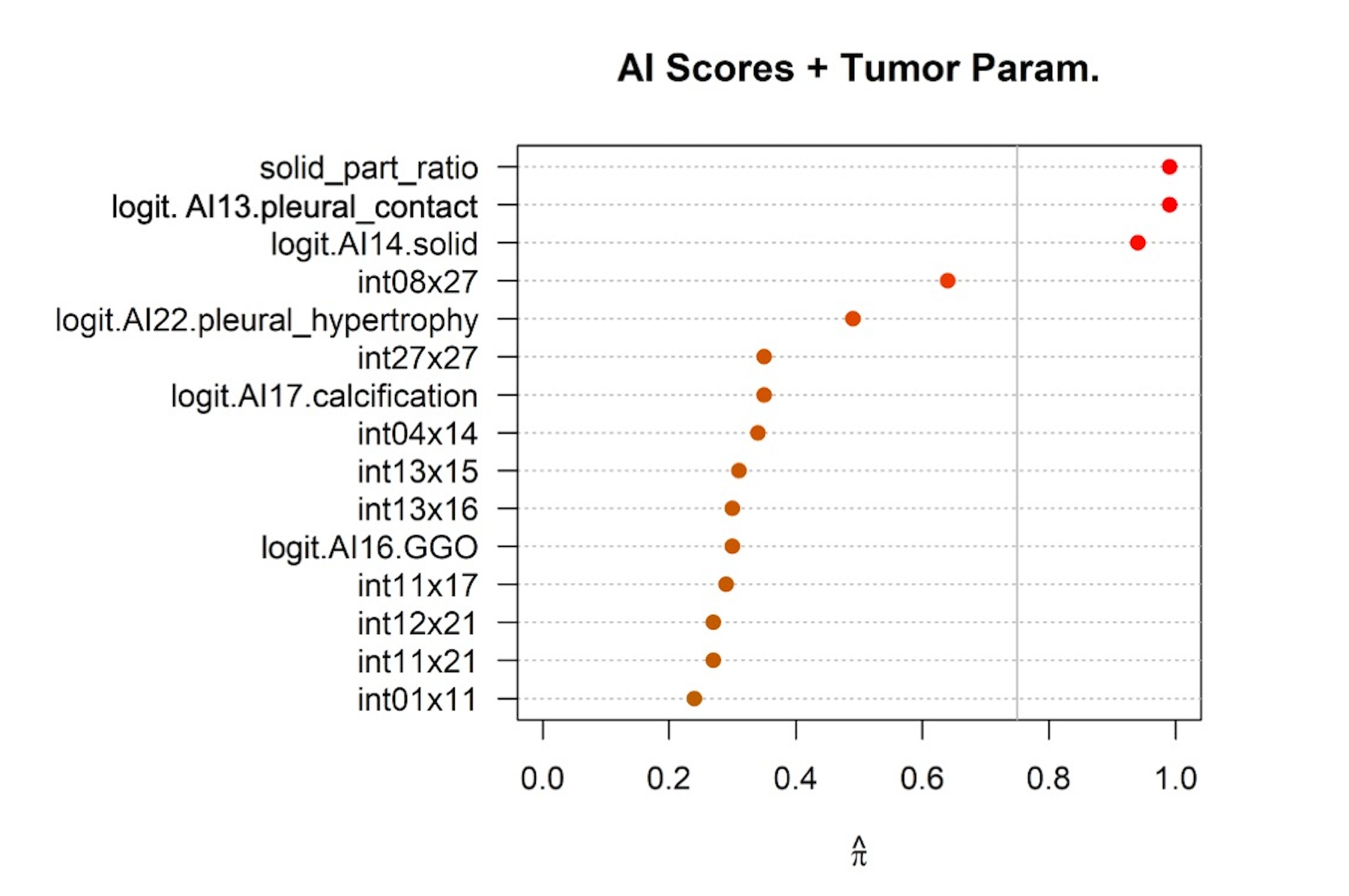

Supplement: S2a Fig — Selection probabilities by the stability selection of model 2. This is the fig S2a legend. Empirical selection probability of each variable by stability selection in model 2. The closer the value on the x-axis is to 1, the more stably the variable is selected across repeated resampling, indicating a high likelihood of association with the target variable. Variables like ‘int01x02’ represent the interaction term of the variables 01 and 02. Please refer to Appendix Table 3 for the variable numbers and their definitions. (TIFF) [file pone.0332956.s003.tiff]

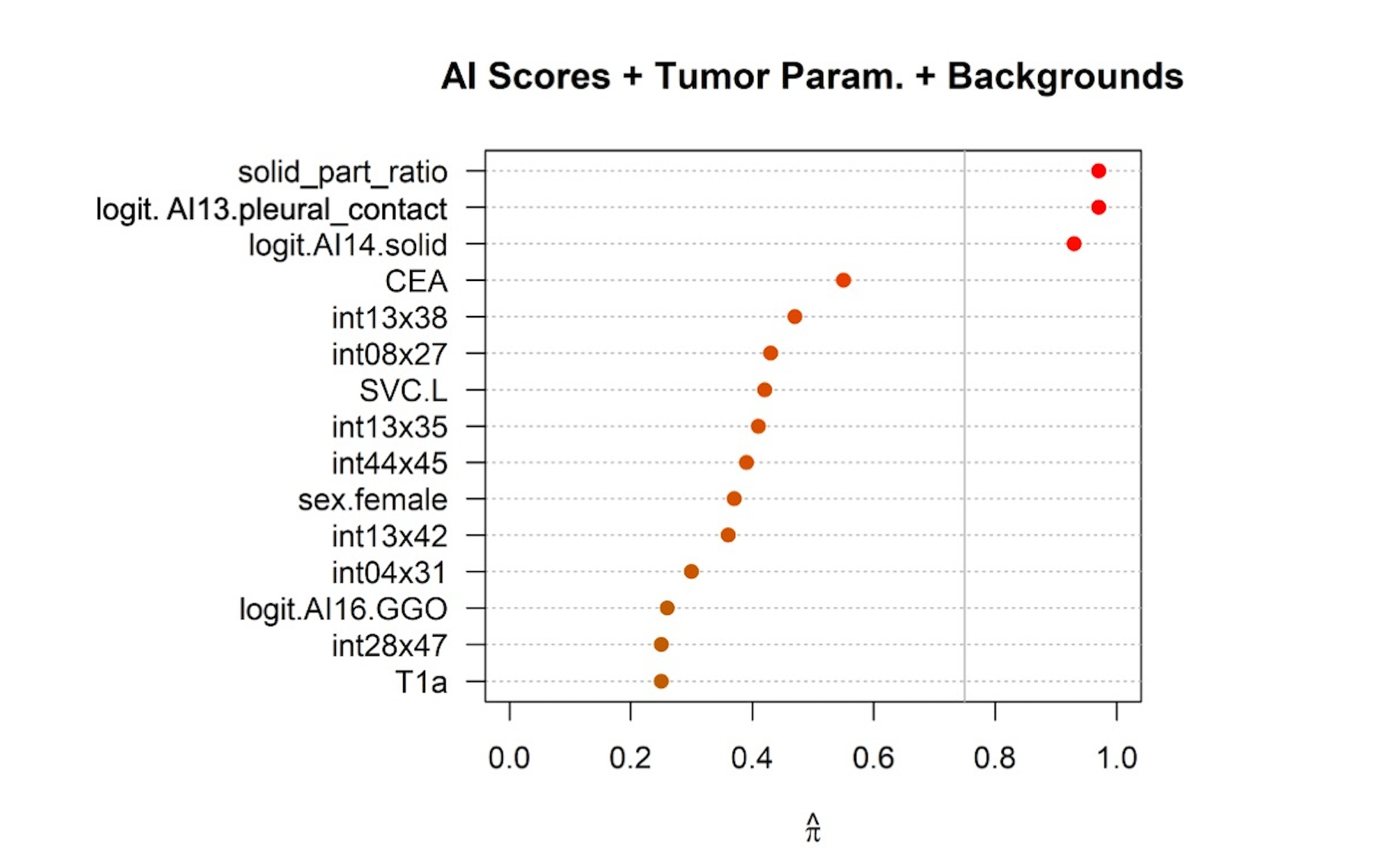

Supplement: S2b Fig — Selection probabilities by the stability selection of model 3. This is the fig S2b legend. Empirical selection probability of each variable by stability selection in model 3. The closer the value on the x-axis is to 1, the more stably the variable is selected across repeated resampling, indicating a high likelihood of association with the target variable. Variables like ‘int01x02’ represent the interaction term of the variables 01 and 02. Please refer to Appendix Table 3 for the variable numbers and their definitions. (TIFF) [file pone.0332956.s004.tiff]

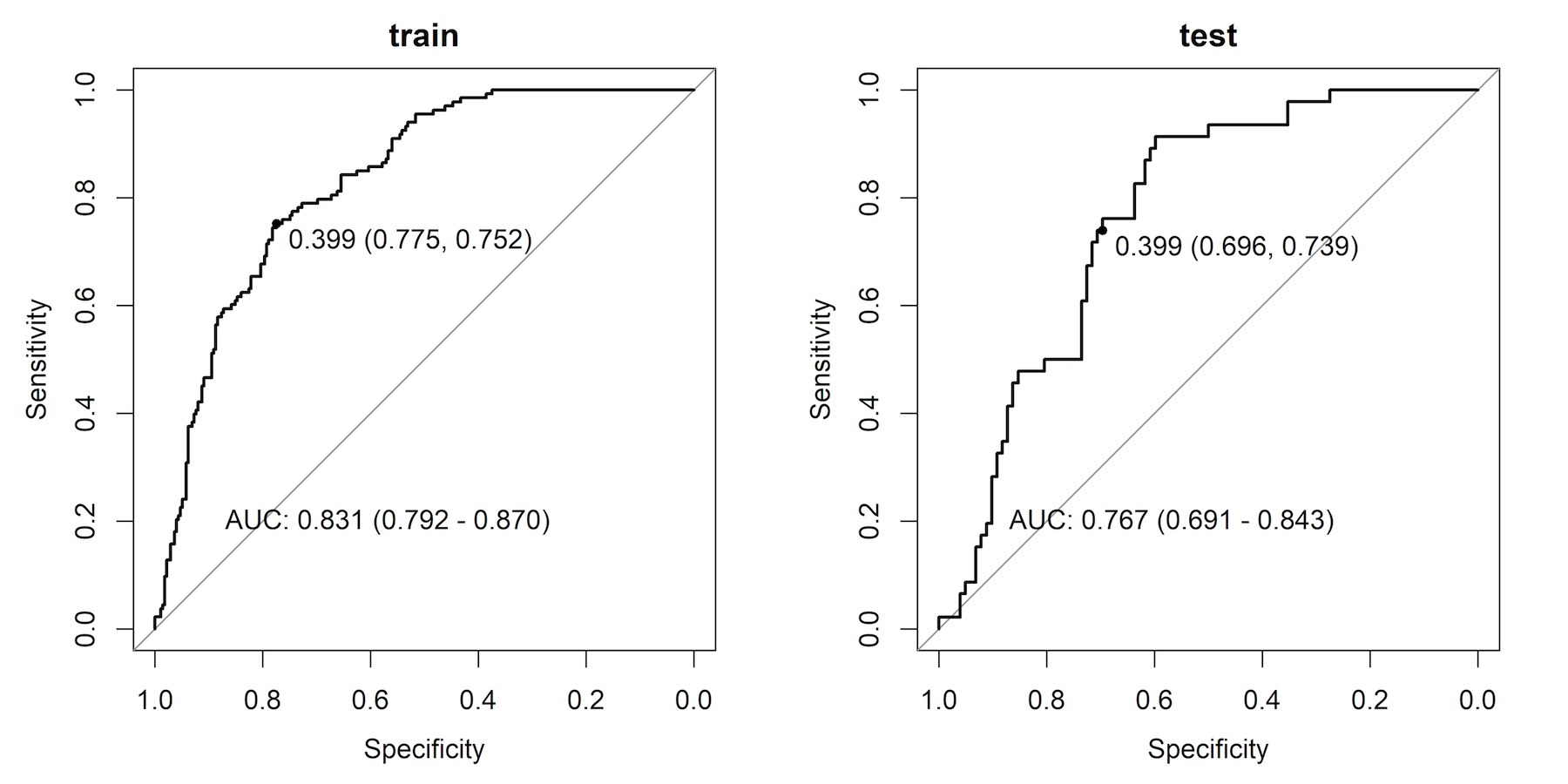

Supplement: S3 Fig — This is the fig S3 legend. The predictive performance of models 2 and 3 was evaluated based on the ROC curve and its area under the curve (AUC), as well as the sensitivity and specificity at the optimal cutoff. The cutoff value of 0.399 was the point in the training cohort where the sum of sensitivity and specificity was the highest, and the sensitivity and specificity in the test cohort were also evaluated using the same cutoff. (TIFF) [file pone.0332956.s005.tiff]

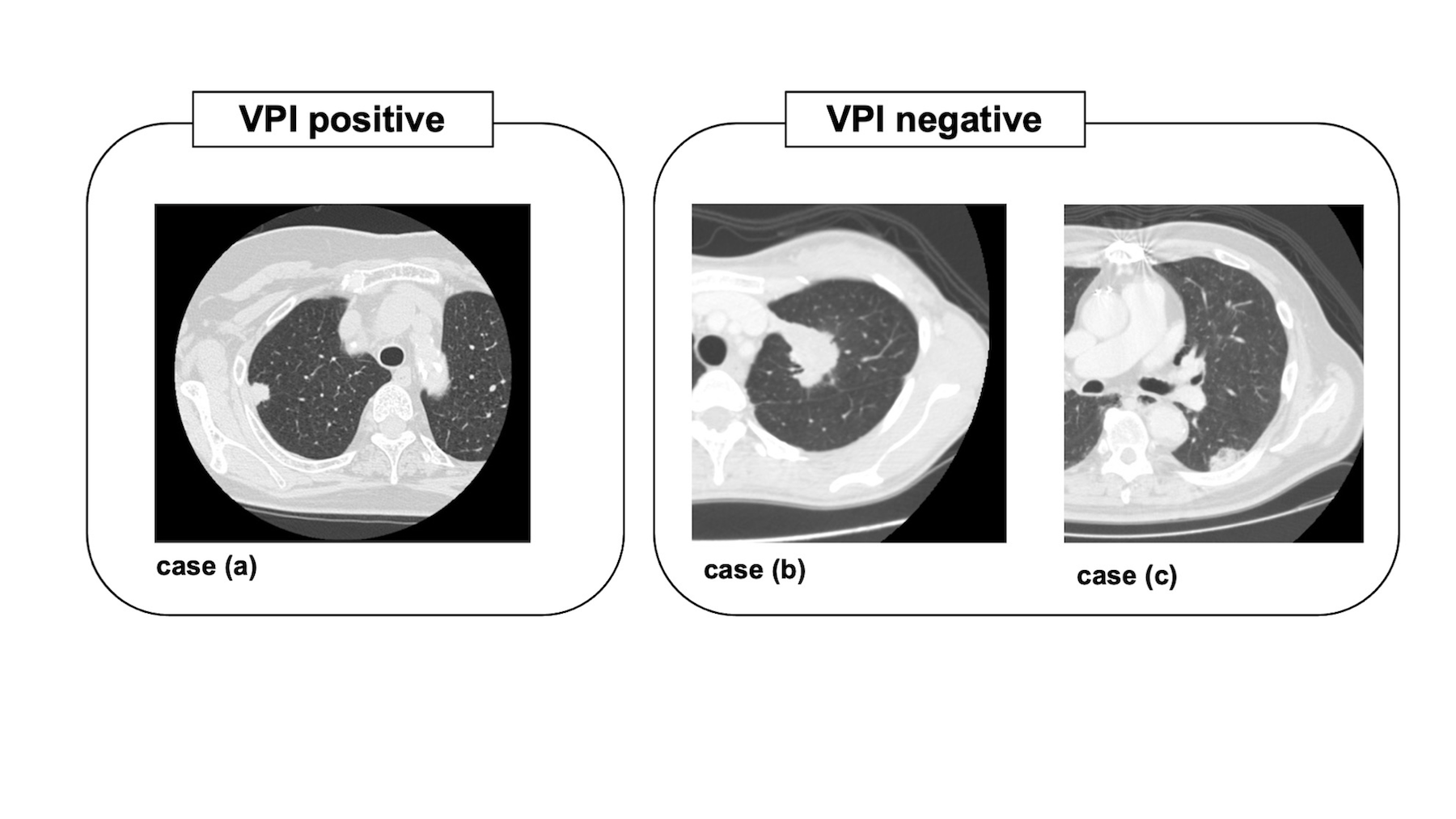

Supplement: S4 Fig — This is the fig S4 legend. Case (a): AI confidence score of “Pleural contact” was 0.99, and “Solid nodule” was 1. This case represented a high “Solid nodule” and “Pleural contact,” showing a high VPI probability score using the prediction model. As predicted, the VPI was positive. Case (b): AI confidence score of “Pleural contact” was 0.34, and “Solid nodule” was 0.908. This case represented a VPI negative case with a high “Solid nodule” score and a low “Pleural Contact” score. The prediction model showed a low VPI probability score. Case (c): AI confidence score of “Pleural contact” was 0.99, and “Solid nodule” was 0.14. This case represented a VPI negative situation with a low “Solid nodule” score and a high “Pleural Contact” score. The prediction model showed a low VPI probability score. (TIFF) [file pone.0332956.s006.tiff]

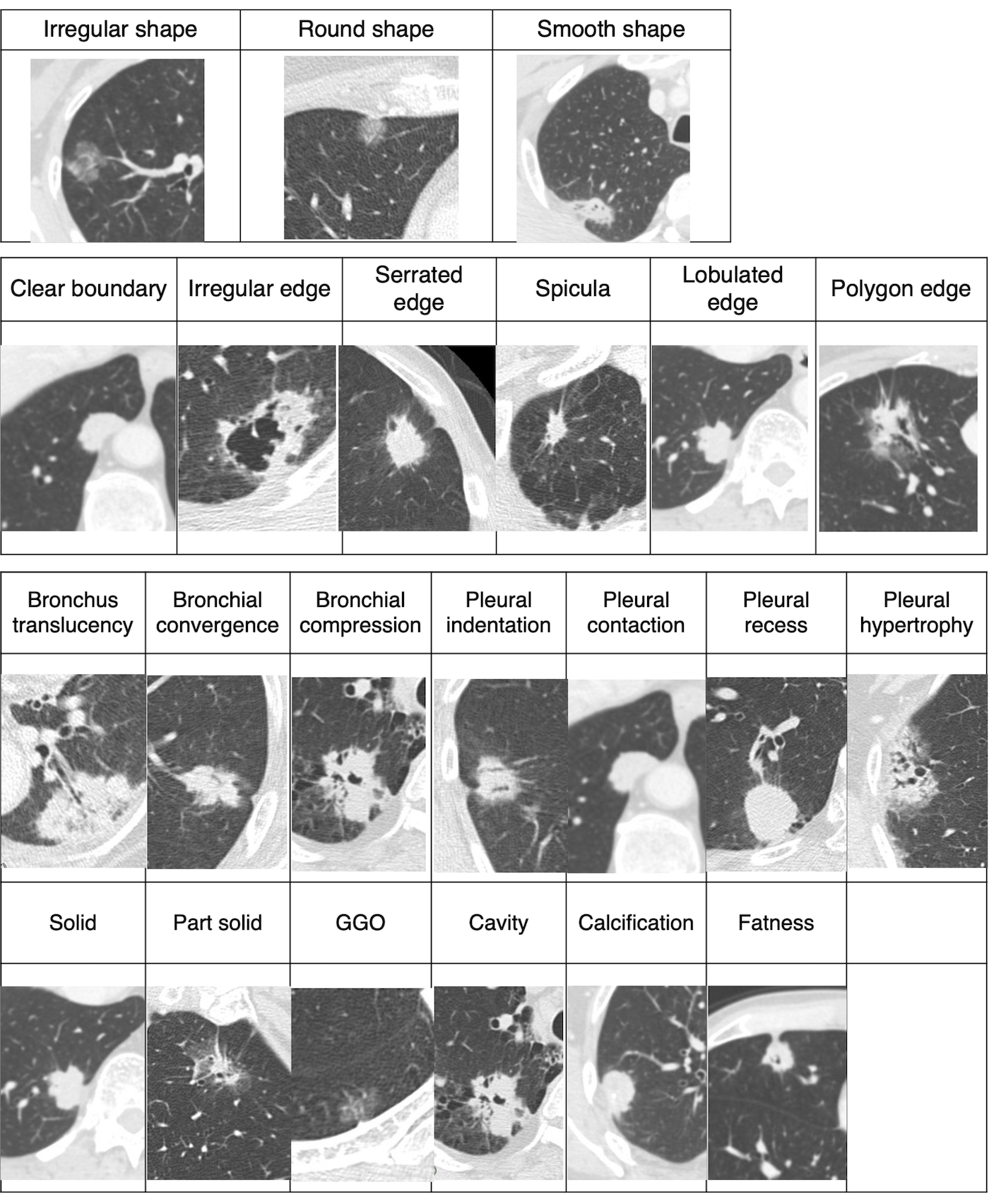

Supplement: S5 Fig — This is the fig S5 legend. This figure illustrates representative CT images demonstrating the 22 radiological features automatically extracted by the AI model. Each panel corresponds to one feature, with visual examples defining each characteristic. (TIFF) [file pone.0332956.s007.tiff]
