# Supplementary material for: AI-driven 3D CT imaging prediction model for improving preoperative detection of visceral pleural invasion in early-stage lung cancer
Source: PLoS One. 2025 Oct 17;20(10):e0332956. doi: 10.1371/journal.pone.0332956 (PMC12533904; doi:10.1371/journal.pone.0332956)
Supplement: S1 Table — (DOCX) [file pone.0332956.s008.docx]

**Table S1. This is the Clinicopathological factors and 3D imaging features.**

| **Variable** | | **Overall (%) (n = 556)** | **Train cohort (%) (n = 408)** |
| --- | --- | --- | --- |
| **No. (%) with data** | | 556 (100) | 408 (100) |
| **Clinicopathological factors** | |  |  |
| **Sex** |  |  |  |
| **Women** | 258 (46.4) | 192 (47.1) |  |
| **Men** | 298 (53.6) | 216 (52.9) |  |
| **Age, median (IQR), years** | 70 (63, 76) | 69 (62, 75) |  |
| **Height, median (IQR), cm** | 160 (153, 167) | 160 (153, 166) |  |
| **Weight, median (IQR), kg** | 58 (51, 66) | 57 (51, 66) |  |
| **Ever smoker** | 340 (61.2) | 240 (58.8) |  |
| **Brinkman index, median (IQR)** | 400 (0, 940) | 300 (0, 940) |  |
| **Past medical history of cardiac disease** | 58 (10.4) | 38 (9.3) |  |
| **Past medical history of respiratory disease** | 159 (28.6) | 105 (25.7) |  |
| **Past medical history of cancer** | 95 (17.1) | 63 (15.4) |  |
| **Pulmonary function test** |  |  |  |
| **SVC, median (IQR), L** | 3.16 (2.63, 3.77) | 3.15 (2.64, 3.76) |  |
| **%SVC, median (IQR), %** | 114 (103, 125) | 114 (102, 126) |  |
| **FEV1, median (IQR), L** | 2.21 (1.85, 2.69) | 2.22 (1.85, 2.71) |  |
| **FEV1%, median (IQR), %** | 74 (68, 79) | 75 (68, 80) |  |
| **Tumor marker** |  |  |  |
| **CEA positive (>5.0 ng/ml)** | 139 (25.0) | 94 (23.0) |  |
| **CYFRA positive (>3.5 ng/ml)** | 49 (8.8) | 32 (7.8) |  |
| **NSE positive (>16.3 ng/ml)** | 13 (2.3) | 9 (2.2) |  |
| **Clinical T factor (8^th^ Ed.)** |  |  |  |
| **Tis** | 13 (2.3) | 10 (2.5) |  |
| **T1mi** | 17 (3.1) | 8 (2.0) |  |
| **T1a** | 76 (13.7) | 58 (14.2) |  |
| **T1b** | 166 (29.9) | 124 (30.4) |  |
| **T1c** | 145 (26.1) | 113 (27.7) |  |
| **T2a** | 139 (25.0) | 95 (23.3) |  |
| **Clinical stage (8th Ed.)** |  |  |  |
| **0** | 13 (2.3) | 10 (2.5) |  |
| **ⅠA1** | 93 (16.7) | 66 (16.2) |  |
| **ⅠA2** | 166 (29.9) | 124 (30.4) |  |
| **ⅠA3** | 145 (26.1) | 113 (27.7) |  |
| **ⅠB** | 139 (25.0) | 95 (23.3) |  |
| **Tumor location** |  |  |  |
| **Left** | 242 (43.5) | 179 (43.9) |  |
| **Right** | 314 (56.5) | 229 (56.1) |  |
| **Tumor location** |  |  |  |
| **Upper lobe** | 324 (58.3) | 242 (59.3) |  |
| **Middle lobe** | 21 (3.8) | 18 (4.4) |  |
| **Lower lobe** | 211 (37.9) | 148 (36.3) |  |
| **Radiological whole tumor size, median (IQR), cm** | 2.3 (1.7, 3.0) | 2.3 (1.8, 3.0) |  |
| **Radiological solid size, median (IQR), cm** | 2.0 (1.3, 2.7) | 2.0 (1.3, 2.7) |  |
| **Surgical procedure** |  |  |  |
| **Lobectomy/segmentectomy** | 525 (94.4) | 391 (95.8) |  |
| **Wedge resection** | 31 (5.6) | 17 (4.2) |  |
| **Pathological whole tumor size, median (IQR), cm** | 2.5 (1.8, 3.4) | 2.5 (1.8, 3.2) |  |
| **Visceral pleural invasion** |  |  |  |
| **PL0** | 377 (67.8) | 275 (67.4) |  |
| **PL1** | 129 (23.2) | 97 (23.8) |  |
| **PL2** | 37 (6.7) | 27 (6.6) |  |
| **PL3** | 13 (2.3) | 9 (2.2) |  |
| **Histology** |  |  |  |
| **Adenocarcinoma** | 464 (83.5) | 340 (83.3) |  |
| **Squamous cell carcinoma** | 65 (11.7) | 48 (11.8) |  |
| **others** | 27 (4.9) | 20 (4.9) |  |
| **Pathological lymph node factor** |  |  |  |
| **N0** | 451 (81.1) | 328 (80.4) |  |
| **N1** | 46 (8.3) | 32 (7.8) |  |
| **N2** | 42 (7.6) | 36 (8.8) |  |
| **NX** | 17 (3.1) | 12 (2.9) |  |
| **Pathological stage (7th Ed.)** |  |  |  |
| **IA** | 255(45.9) | 63(42.6) |  |
| **IB** | 180(32.4) | 56(37.8) |  |
| **II-IV** | 121(21.8) | 29(19.6) |  |
| **3D imaging features** |  |  |  |
| **Radiological whole tumor volume (AI software), median (IQR), mm3** | 3,741 (1,782, 6,762) | 3,887 (1,826, 6,834) |  |
| **Radiological GGN-part volume (AI software), median (IQR), mm3** | 1,092 (476, 1,983) | 1,103 (514, 1,977) |  |
| **Radiological GGN-part volume ratio (AI software), median (IQR)** | 31 (14, 65) | 30 (14, 61) |  |
| **Radiological solid-part volume (AI software), median (IQR), mm3** | 1,899 (671, 4,556) | 2,017 (797, 4,662) |  |
| **Radiological solid-part volume ratio (AI software), median (IQR)** | 69 (35, 86) | 70 (39, 86) |  |

Ed, Edition; PL, Pleural invasion; GGN, Ground-glass nodule
