# Supplementary material for: AI-driven 3D CT imaging prediction model for improving preoperative detection of visceral pleural invasion in early-stage lung cancer
Source: PLoS One. 2025 Oct 17;20(10):e0332956. doi: 10.1371/journal.pone.0332956 (PMC12533904; doi:10.1371/journal.pone.0332956)
Supplement: S2 Table — (DOCX) [file pone.0332956.s009.docx]

**Table S2. This is the Association between clinicopathologic factors and VPI in the training cohorts.**

| **Variable** | **Overall** **(%)** (n = 408) | **Non-VPI** **(%)** (n = 275) | **VPI** **(%)** (n = 133) | **p-value**^a^  (non-VPI vs VPI) |
| --- | --- | --- | --- | --- |
| **No. (%) with data** | 408 (100) | 275 (100) | 133 (100) |  |
| **Sex, Men** | 216 (52.9) | 121 (44.0) | 95 (71.4) | <0.001 |
| **Age, median (IQR), years** | 69 (62, 75) | 68 (62, 75) | 69 (62, 75) | 0.732 |
| **Ever smoker** | 240 (58.8) | 140 (50.9) | 100 (75.2) | <0.001 |
| **Tumor location, Right** | 229 (56.1) | 155 (56.4) | 74 (55.6) | 0.890 |
| **Radiological whole tumor size, median (IQR), cm** | 2.3 (1.8, 3.0) | 2.2 (1.7, 3.0) | 2.4 (1.9, 3.1) | 0.075 |
| **Radiological solid size, median (IQR), cm** | 2.0 (1.3, 2.7) | 1.8 (1.0, 2.5) | 2.4 (1.9, 3.0) | <0.001 |
| **Surgical procedure: Lobectomy / Segmentectomy** | 391 (95.8) | 267 (97.1) | 124 (93.2) | 0.068 |
| **Pathological whole tumor size, median (IQR), cm** | 2.5 (1.8, 3.2) | 2.4 (1.7, 3.0) | 2.8 (2.2, 3.5) | <0.001 |
| **Histology, Adenocarcinoma** | 340 (83.3) | 246 (89.5) | 94 (70.7) | <0.001 |
| **Lymph node metastasis** | 68 (16.7) | 27 (9.8) | 41 (30.8) | <0.001 |
| ^a^Wilcoxon rank sum test; Pearson's Chi-squared test; Fisher's exact test | | | | |
