# Supplementary material for: AI-driven 3D CT imaging prediction model for improving preoperative detection of visceral pleural invasion in early-stage lung cancer
Source: PLoS One. 2025 Oct 17;20(10):e0332956. doi: 10.1371/journal.pone.0332956 (PMC12533904; doi:10.1371/journal.pone.0332956)
Supplement: S3 Table — (DOCX) [file pone.0332956.s010.docx]

**Table S3. Relationship between VPI and AI-Derived 22 Radiological Features in the test cohorts**

| Variables | Variable numbers | Overall, n = 148^a^ | Non-VPI, n = 102^a^ | VPI, n = 46^a^ | AUC (95% CI) |
| --- | --- | --- | --- | --- | --- |
| Clear boundary | 1 | 0.239 (0.041, 0.727) | 0.088 (0.030, 0.606) | 0.645 (0.265, 0.826) | 0.711 (0.623 - 0.799) |
| Irregular shape | 2 | 0.996 (0.981, 0.999) | 0.996 (0.979, 0.999) | 0.997 (0.984, 0.999) | 0.501 (0.402 - 0.599) |
| Round shape | 3 | 0.002 (0.000, 0.017) | 0.002 (0.000, 0.020) | 0.003 (0.001, 0.015) | 0.507 (0.409 - 0.605) |
| Smooth shape | 4 | 0.000 (0.000, 0.001) | 0.000 (0.000, 0.001) | 0.000 (0.000, 0.001) | 0.493 (0.394 - 0.592) |
| Irregular edge | 5 | 0.987 (0.922, 0.998) | 0.989 (0.927, 0.999) | 0.985 (0.901, 0.996) | 0.557 (0.460 - 0.654) |
| Serrated edge | 6 | 0.818 (0.191, 0.957) | 0.619 (0.095, 0.942) | 0.906 (0.771, 0.974) | 0.674 (0.585 - 0.762) |
| Spiculation | 7 | 0.770 (0.302, 0.931) | 0.703 (0.228, 0.923) | 0.897 (0.567, 0.935) | 0.619 (0.525 - 0.713) |
| Lobulated edge | 8 | 0.634 (0.311, 0.876) | 0.557 (0.234, 0.840) | 0.769 (0.566, 0.942) | 0.669 (0.580 - 0.759) |
| Polygon edge | 9 | 0.019 (0.008, 0.044) | 0.021 (0.008, 0.059) | 0.014 (0.007, 0.028) | 0.590 (0.494 - 0.686) |
| Bronchus translucency | 10 | 0.341 (0.060, 0.950) | 0.459 (0.089, 0.970) | 0.122 (0.025, 0.526) | 0.667 (0.574 - 0.761) |
| Cavity | 11 | 0.017 (0.006, 0.093) | 0.018 (0.006, 0.080) | 0.008 (0.005, 0.109) | 0.533 (0.429 - 0.638) |
| Pleural indentation | 12 | 0.757 (0.353, 0.969) | 0.830 (0.376, 0.979) | 0.611 (0.295, 0.935) | 0.589 (0.496 - 0.682) |
| Pleural contact | 13 | 0.959 (0.326, 0.995) | 0.744 (0.178, 0.990) | 0.989 (0.954, 0.997) | 0.697 (0.609 - 0.786) |
| Solid | 14 | 0.803 (0.000, 1.000) | 0.004 (0.000, 1.000) | 1.000 (0.989, 1.000) | 0.741 (0.658 - 0.824) |
| Part solid | 15 | 0.007 (0.000, 0.987) | 0.387 (0.000, 0.996) | 0.000 (0.000, 0.011) | 0.700 (0.610 - 0.789) |
| GGO | 16 | 0.000 (0.000, 0.002) | 0.000 (0.000, 0.007) | 0.000 (0.000, 0.000) | 0.732 (0.650 - 0.814) |
| Calcification | 17 | 0.024 (0.015, 0.044) | 0.021 (0.013, 0.032) | 0.039 (0.022, 0.052) | 0.692 (0.605 - 0.780) |
| Fatness | 18 | 0.000 (0.000, 0.001) | 0.000 (0.000, 0.001) | 0.001 (0.000, 0.002) | 0.587 (0.490 - 0.685) |
| Bronchial convergence | 19 | 0.925 (0.823, 0.975) | 0.930 (0.843, 0.979) | 0.920 (0.746, 0.955) | 0.584 (0.489 - 0.680) |
| Bronchial compression | 20 | 0.027 (0.014, 0.075) | 0.021 (0.010, 0.053) | 0.044 (0.020, 0.103) | 0.672 (0.580 - 0.764) |
| Pleural recess | 21 | 0.885 (0.675, 0.959) | 0.878 (0.679, 0.964) | 0.886 (0.662, 0.944) | 0.470 (0.373 - 0.567) |
| Pleural hypertrophy | 22 | 0.743 (0.347, 0.845) | 0.730 (0.285, 0.830) | 0.761 (0.601, 0.863) | 0.579 (0.484 - 0.675) |

^a^Median (IQR); n (%)

GGO, Ground-glass opacity
