# Supplementary material for: AI-driven 3D CT imaging prediction model for improving preoperative detection of visceral pleural invasion in early-stage lung cancer
Source: PLoS One. 2025 Oct 17;20(10):e0332956. doi: 10.1371/journal.pone.0332956 (PMC12533904; doi:10.1371/journal.pone.0332956)
Supplement: Attachment — Submitted filename: Reviewer_comments.docx [file pone.0332956.s011.docx]

**Summary**

The authors have developed and validated a model to detect visceral pleural invasion (VPI) in early-stage non-small cell lung cancer (NSCLC) using AI-assisted 3D CT imaging, employing a commercial software, which is easily accessible. The model demonstrates promising diagnostic performance (AUC nearly 0.8) based on features such as "solid nodule" and "pleural contact." However, several issues must be addressed to improve both scientific rigor and clinical applicability.

**Major Strengths**

The study presents a technically robust approach to a clinically meaningful problem. By focusing on AI-assisted 3D CT imaging, the authors overcome limitations inherent to conventional 2D imaging and subjective radiological interpretation. In particular, 3D deep learning-derived features of various cancers can be the “next radiomics” features as they are now easily implemented and accessible not only across cancer types but also across modality (i.e. CT and MR). It would be appreciated if the authors consider to cite the following paper by Lee and Ahn et al. (Neuro-Onc, 2024) as one of the previous studies of AI-driven 3D imaging features for other types of cancers: <https://pubmed.ncbi.nlm.nih.gov/37855826/>.

The use of objective, algorithmically extracted features such as "solid nodule" and "pleural contact" enhances reproducibility, and the application of stability selection and regularized logistic regression adds methodological rigor.

**Major Weaknesses**

Despite the strong methodological framework, several limitations reduce the immediate clinical applicability of the model.

1. The lack of external validation raises concerns about generalizability across institutions and imaging settings. The authors are encouraged to do external validation or if not possible, at least 5-fold cross validation to provide the generalized performance of the developed model.
2. The model was developed exclusively on tumors‚ larger than 4 cm and in pleural contact, potentially introducing selection bias and limiting applicability to the broader NSCLC population. Please provide the references to justify this exclusion criteria.
3. Table 2 seemed to provide the comparison with radiologist performance or assessment with the model performance. However, these results can be more emphasized and elaborated in the Discussion.

**Minor Issues**

1. The resolution of Figure 1B, which is crucial, is too low to recognize. Please submit the figure as high resolution as possible. Are there any attention maps such as Grad-CAM, which is provided by the software?
2. The term "pleural contract" appears to be a typographical error and should be corrected to "pleural contact" (e.g., Table 2, page 20).

**Recommendation**

This is a valuable and technically well-executed study with clear clinical potential. However, given the aforementioned limitations-particularly the lack of external validation, unclear clinical integration, and limited generalizability-a Major Revision is recommended.
