# Supplementary material for: AI-driven 3D CT imaging prediction model for improving preoperative detection of visceral pleural invasion in early-stage lung cancer
Source: PLoS One. 2025 Oct 17;20(10):e0332956. doi: 10.1371/journal.pone.0332956 (PMC12533904; doi:10.1371/journal.pone.0332956)
Supplement: Attachment — Submitted filename: Response to Reviewers_Nagase et al.PlosOne ver.3.docx [file pone.0332956.s013.docx]

**POINT-BY-POINT RESPONSES TO REVIEWERS**

We thank the reviewers for their positive feedback and constructive comments and suggestions. We provide below our point-by-point responses to the comments, and our explanations as to how they were addressed in the revised manuscript. Revised sentences in the manuscript have been highlighted in red.

**Reviewer #1:**

**Major Strengths:**

**Comment 1:**

It would be appreciated if the authors consider to cite the following paper by Lee and Ahn et al. (Neuro-Onc, 2024) as one of the previous studies of AI-driven 3D imaging features for other types of cancers: <https://pubmed.ncbi.nlm.nih.gov/37855826/>.

**Response 1:**

Thank you so much for the helpful comment. As the reviewer suggested, we have added the recommended article for citation as follows.

(Line 74, page 6 )

Recently, AI has significantly contributed to advancements in the medical field (18), assisting in highly precise evaluation of radiological diagnoses and providing objective radiological findings (19,20).

**Major Weaknesses:**

**Comment 1:**

**The lack of external validation raises concerns about generalizability across institutions and imaging settings. The authors are encouraged to do external validation or if not possible, at least 5-fold cross validation to provide the generalized performance of the developed model.**

**Response1:**
Thank you for your valuable comments. We have revised the Methods section accordingly.

As you pointed out, verifying the generalizability across institutions and image settings is critically important and remains a limitation of this study. Nevertheless, we implemented certain procedures to evaluate external validity in our model assessment.

The performance of the model developed in this study was evaluated using a test cohort that was not involved in the training process. Importantly, the split between the training and test cohorts was not randomized; instead, it was based on the date of surgery (June 1, 2017)._This temporal split allows us to assess a degree of external validity in terms of the period during which patients received care, in contrast to conventional cross-validation based on random partitioning.

This approach is referred to as "temporal validation” and is described in the TRIPOD statement as a method that lies between internal and external validation.

(Line 105, page 8)

**Fig 1. Flow chart.** Out of the total 556 patients, 408 patients, were assigned to the training cohort, and the other 148 patients were assigned to the test cohort at a ratio of 3:1 based on the date of surgery (June 1, 2017). For model construction, three sets of candidate variables, including the AI confidence scores, other tumor parameters, and patient characteristics, were prepared.

(Line 169, page 13)

Using the stability selection algorithm with L1-regularized logistic regression (Lasso), we selected important features associated with VPI among the AI-derived features and certain patient characteristics. The variable selection algorithm was applied to three nested sets of features: AI confidence score alone, AI confidence score with 3D radiological parameters, and patient characteristics. All interaction terms were included. Using the selected features for each set, we performed logistic regression modeling to develop a VPI prediction model. Variable selection and model development were performed for the training cohort, and model evaluation was performed for the test cohort.

Variable selection methods such as stepwise regression and Lasso are highly sensitive to sample variability, and their results may not be stable. In this study, we employed the stability selection algorithm to identify factors that are likely to be associated with VPI. The stability selection algorithm repeatedly applies a variable selection method to subsampled datasets and evaluates the selection probability of each feature. This approach identifies features that are more likely to be truly associated with the outcome variable (30, 31).

**Comment 2:**

**The model was developed exclusively on tumors‚ larger than 4 cm and in pleural contact, potentially introducing selection bias and limiting applicability to the broader NSCLC population. Please provide the references to justify this exclusion criteria.**

**Response 2:**

Thank you for your insightful comment. We agree that it is important to clarify the background for our cohort selection.

We limited our analysis to tumors ≤4 cm for two main reasons:

1.	According to the 8th TNM classification, VPI changes stage only in tumors ≤4 cm (e.g., from stage IA to IB). Tumors >4 cm are stage II or higher regardless of VPI, making VPI prediction less clinically relevant in larger tumors.

2.	Previous studies (1-3) have shown that in tumors >3 cm, VPI does not significantly impact prognosis or lymph node metastasis.

We have clarified this rationale and added the appropriate references in the revised manuscript.

(Supplementary Methods, Line 11, page 1)

We excluded patients with tumors larger than 4 cm in size, as these are classified as stage II or higher according to the 8th TNM classification and VPI does not alter their staging. In addition, previous studies have shown that in tumors >3 cm, VPI has limited impact on prognosis and nodal metastasis (1-3).

**Comment 3:**

**Table 2 seemed to provide the comparison with radiologist performance or assessment with the model performance. However, these results can be more emphasized and elaborated in the Discussion.**

**Response 3:**

Thank you for your suggestion to further emphasize and elaborate on the results shown in Table 2. We have revised the Discussion to highlight the diagnostic performance of key AI-derived features and to clarify the multivariate analysis process used to identify independent predictors of VPI. The revised text now specifies that we evaluated three different multivariate modeling approaches—stepwise selection, backward elimination, and forward selection—but obtained inconsistent results. Therefore, we applied the stability selection method, which performs stable variable selection on a multivariate logistic regression model including all candidate variables and their interaction terms. This approach identified two features as the most robust predictors, and the results can be interpreted as those from a multivariate logistic regression. We believe this addition improves the clarity and reproducibility of our statistical analysis.

(Line 387, page 35)

In addition, our study analyzed three models that included a multitude of variables ranging from clinical data and 3D tumor volume measurements to 22 radiological features. Multivariate analysis using stepwise, backward, and forward selection yielded different results, so we applied stability selection, which identifies robust predictors in a multivariate logistic regression framework. Two features, “Solid nodule” and” Pleural contract,” remained.

**Minor Issues:**

**Comment 1:**

**The resolution of Figure 1B, which is crucial, is too low to recognize. Please submit the figure as high resolution as possible. Are there any attention maps such as Grad-CAM, which is provided by the software?**

**Response 1:**

We appreciate the reviewer’s valuable feedback. We have replaced the Figure with a higher-resolution version to ensure that it is clear and easily interpretable. The Synapse Vincent AI software also uses a simplified VGG-based CNN with 12 3D-convolutional layers and two max pooling layers. Grad-CAM or similar attention maps are not included in its standard output and were not used in this study.

(Lines 141-144, page 11)

As described in the cited study, this algorithm uses a CNN model that is a simplified version of the VGG architecture with 12 three-dimensional convolutional layers and two max pooling layers (26). A screenshot of the analytical process is shown (Fig 2).

**Comment 2:**

**The term "pleural contract" appears to be a typographical error and should be corrected to "pleural contact" (e.g., Table 2, page 20).**

**Response 2:**

We appreciate the reviewer’s valuable comment. We have carefully reviewed the manuscript and corrected all instances of “pleural contract” to “pleural contact”.

**Reviewer #2:**

**Comment 1:**

**The significant differences in age and pathological tumor size observed between the training and test cohorts highlight the need for cross-validation to confirm the robustness and generalizability of the developed model.**

**Response 1:**

Thank you for your thoughtful comments.

Unlike comparisons between treatment and control groups in randomized controlled trials, it is generally not problematic for the distributions of covariates to differ between the training and test cohorts.

As also noted in our response to Reviewer #1, the split between the training and test cohorts in this study was not randomized but instead based on the date of surgery (June 1, 2017). Accordingly, the model's performance on the test cohort reflects external validity in a temporal sense, which we consider to be a more appropriate assessment of generalizability than conventional cross-validation.

This approach is referred to as **"temporal validation"**, and is described in the TRIPOD statement as a method that lies between internal and external validation.

Although the model’s performance declined in the test cohort compared to the training cohort, we believe this reflects not only potential overfitting but also changes in patient characteristics over time. From the perspective of evaluating the model’s utility, we consider the performance observed in the test cohort, which is composed of more recent patients, to be more informative and clinically relevant.

(Line 169, page 13)

Using the stability selection algorithm with L1-regularized logistic regression (Lasso), we selected important features associated with VPI among the AI-derived features and certain patient characteristics. The variable selection algorithm was applied to three nested sets of features: AI confidence score alone, AI confidence score with 3D radiological parameters, and patient characteristics. All interaction terms were included. Using the selected features for each set, we performed logistic regression modeling to develop a VPI prediction model. Variable selection and model development were performed for the training cohort, and model evaluation was performed for the test cohort.

Variable selection methods such as stepwise regression and Lasso are highly sensitive to sample variability, and their results may not be stable. In this study, we employed the stability selection algorithm to identify factors that are likely to be associated with VPI. The stability selection algorithm repeatedly applies a variable selection method to subsampled datasets and evaluates the selection probability of each feature. This approach identifies features that are more likely to be truly associated with the outcome variable (30, 31).

**Comment 2:**

**The statistical analysis presented in Table 2 appears incomplete. It is recommended to perform both univariate and multivariate logistic regression analyses, complemented by AUC values, to identify and validate independent predictors of vascular invasion (VPI).**

**Response 2:**

Thank you for your comment.

We attempted multivariate logistic regression using several approaches, including models with all variables included, stepwise selection, and straightforward application of Lasso regression. However, the results varied considerably depending on the method, making interpretation difficult. We suspect that this variability was due to high correlations among the AI features, leading to multicollinearity.

Given this, we employed stability selection to construct a model that includes variables more likely to be robustly associated with VPI. We have expanded the description of the stability selection procedure, including our rationale for its use, in the Methods section.

Stability selection, roughly speaking, performs stable variable selection on a multivariate logistic regression model that includes all candidate variables and their interaction terms. Therefore, the results may be interpreted as those from a multivariate logistic regression.

(Line 387, page 35)

In addition, our study analyzed three models that included a multitude of variables ranging from clinical data and 3D tumor volume measurements to 22 radiological features. Multivariate analysis using stepwise, backward, and forward selection yielded different results, so we applied stability selection, which identifies robust predictors in a multivariate logistic regression framework. Two features, “Solid nodule” and ”Pleural contract,” remained.

**Comment 3:**

**The table titles in the manuscript lack clarity. To improve understanding: Table 1 should explicitly specify that it compares baseline characteristics between the cohorts, e.g., "Baseline Characteristics: Training versus Test Cohorts."**

**Table 3 should have a more descriptive title, such as "Multivariable Logistic Regression Models for VPI Prediction."**

**Response 3:**

We thank the reviewer for their valuable comment. We have changed the titles of the table as follows:

(Line 228, page 16)

Table 1. Baseline Characteristics: Training versus Test Cohorts.

(Line 285, page 27)

Table 3. Multivariable Logistic Regression Models with L1 regularization for VPI Prediction.

**Comment 4:**

**A DeLong test should be conducted to perform a statistical comparison between the ROC curves derived from the training and test cohorts, providing a quantitative assessment of their differences.**

**Response 4:**

Thank you for your comment.

It is true that the DeLong test is commonly used for comparing AUCs. However, we believe that its application in our study is not appropriate for the following three reasons.

First, the null hypothesis of the DeLong test here is that the AUCs from the training and test cohorts are equal. In theory, however, some degree of overfitting is inevitable regardless of the modeling approach used. As a result, this null hypothesis is always false, and conducting a statistical test under such a condition lacks interpretability.

Second, the DeLong test is primarily designed for comparing the performance of different biomarkers or models evaluated on the **same** set of individuals. In contrast, our training and test cohorts are based on different patients, and thus the test is not applicable in this context.

Third—and most importantly—rather than testing for differences between the training and test cohorts, what truly matters is the performance of the model in the test cohort, which includes new patients not used in model development. This reflects not only potential overfitting but also some extent of generalizability of the model. Therefore, we consider the AUC and its confidence interval in the test cohort to be of primary importance.

**Comment 5:**

**In the introduction section (lines 61–63), the critique regarding the limitations of 2D CT imaging would benefit from citing comparable 3D AI studies. Including and cite references such as the study with DOI: 10.1016/j.crad.2021.11.008 would strengthen the literature context.**

**Response 5:**

Thank you so much for the helpful comment. As the reviewer suggested, we have added the recommended article for citation as follows.

(Line 64, page 6)

Radiological assessment using computed tomography (CT) has identified features correlated with VPI, including pleural tags or indents, tumor dimensions and composition, and pleural thickness (7-16).

**Comment 6:**

**Volumetric parameters, such as the solid-part volume ratio, show high odds ratios but were excluded from the final predictive model without justification. Clarifying the rationale for their exclusion is essential.**

**Response 6:**

Thank you for your comment.

Variable selection was automated using the stability selection procedure. Please refer to the Methods section of the revised version for a detailed description of the algorithm.

We suspect that the reason volumetric parameters were excluded is their high correlations with other variables. This implies that the information conveyed by the volumetric parameters was likely captured by other features. For example, the solid-part volume ratio shows high correlations with multiple other variables, as listed below. Such strong correlations can lead to multicollinearity, which undermines the stability of variable selection.

To address this, we employed stability selection to robustly identify variables that are more likely to be truly associated with the outcome.

Highly correlated variables with the solid-part volume ratio:
Clear boundary (AI feature): 0.81
Solid (AI feature): 0.73
Serrated edge (AI feature): 0.72
GGO (AI feature): -0.73

(Line 177, page 13)

Variable selection methods such as stepwise regression and Lasso are highly sensitive to sample variability, and their results may not be stable. In this study, we employed the stability selection algorithm to identify factors that are likely to be associated with VPI. The stability selection algorithm repeatedly applies a variable selection method to subsampled datasets and evaluates the selection probability of each feature. This approach identifies features that are more likely to be truly associated with the outcome variable (28, 29). In our analysis, the upper bound for the expected number of false discoveries was set to one, the cutoff for the selection probability was set to 0.75, and the number of subsampling replicates was set to 50.

**Comment 7:**

**The manuscript mentions 22 radiological features derived via AI; however, these features are not listed. Including a figure and detailed description of these features would enhance clarity and facilitate reproducibility.**

**Response 7:**

We appreciate the reviewer’s insightful comment. We have revised the manuscript to include a detailed description of the 22 AI-derived radiological features used in the analysis. To enhance clarity and reproducibility, we have also added a supplement figure (Figure S5) illustrating representative examples of each feature. We have created a categorized list of the features and added it to the supplemental method.

(Supplementary Methods, Line 65, page 5)

This AI lung nodule analysis model uses a convolutional neural network based on VGG-16 and consists of 12 convolution layers, with four layers removed from the output side of the VGG-16. To extract 3D imaging features, 3D convolution was used for all convolution layers.

A detailed description and figure of the 22 AI-derived radiological features used in the analysis is provided below (Fig S5). These features were automatically extracted from CT images using the AI algorithm. Following the methodology described in reference (7), representative image examples with high AI confidence scores were selected for each of the 22 features.

**Irregular shape**: Tumors with an asymmetrical or uneven contour.

**Round shape**: Tumors with a generally circular or oval outline.

**Smooth shape**: Tumors with a uniformly flat and regular surface.

**Clear boundary**: The tumor surface is distinctly visible and can be clearly differentiated from the surrounding lung parenchyma.

**Serrated edge**: Tumor edge exhibits a saw-tooth-like appearance.

**Spiculation**: Thin lines extending from the edge of the tumor into the surrounding lung tissues.

**Lobulated edge**: Tumor border appears multilobulated.

**Polygon edge**: Tumor outline displays multiple straight segments forming polygonal shapes.

**Bronchus translucency**: Presence of bronchial air passages visible within the tumor.

**Bronchial convergence**: Bronchi appear to converge toward the tumor mass.

**Bronchial compression**: Narrowing of bronchi due to tumor pressure.

**Pleural indentation**: Local retraction of the pleura toward the tumor.

**Pleural contaction**: Tumor contact to the pleural surfaces.

**Pleural recess**: Tumor extension into the pleural recesses.

**Pleural hypertrophy**: Thickening of pleura adjacent to the tumor.

**Solid**: Tumor appears completely opaque without air or fluid components.

**Part-solid**: Tumor contains both solid and ground-glass components.

**Ground-glass opacity (GGO)**: Tumor area with hazy increased attenuation that does not obscure underlying structures.

**Cavity**: Air-filled space within the tumor mass.

**Calcification**: Presence of high-density foci within the tumor indicating calcified material.

**Fat component**: Tumor shows areas with fat-density attenuation.

(Supplementary Methods, Line 204, page 13)

**Fig S5. This is the CT images of representative examples of 22 AI-derived radiological features used in the analysis.** This is the fig S5 legend. This figure illustrates representative CT images demonstrating the 22 radiological features automatically extracted by the AI model. Each panel corresponds to one feature, with visual examples defining each characteristic.

**Reviewer #3:**

**Comment 1:**

**Please revise “spicula” throughout the manuscript to the correct term “spiculation.”**

**Response 1:**

We thank the reviewer for the insightful comment and for identifying the incorrect term. Following the suggestion, we have revised all instances of “spicula” to the appropriate term “spiculation” throughout the manuscript.

**Comment 2:**

**Partial resection” should be corrected to the standard term “wedge resection.” This improves clinical clarity and aligns with surgical literature conventions.**

**Response 2:**

We appreciate the reviewer’s valuable suggestion. To enhance clinical clarity and ensure consistency with established surgical terminology, we have replaced the term “partial resection” with “wedge resection” throughout the manuscript.

**Comment 3:**

**The clinical impact of preoperative VPI detection is insufficiently justified. Since VPI is ultimately diagnosed on pathology, the value of preoperative prediction needs clearer articulation. Please explain why such prediction meaningfully changes surgical planning or postoperative management, especially considering that adjuvant treatment decisions can be made after pathological evaluation.**

**Response 3:**

We thank the reviewer for this important comment and the opportunity to further clarify the clinical significance of preoperative VPI prediction.

We have revised the Introduction to emphasize that VPI is strongly associated with lymph node metastasis, which has direct implications for surgical planning, particularly in the current era where segmentectomy is increasingly performed for small-sized NSCLC. Because lymph node metastasis generally excludes eligibility for segmentectomy, and intraoperative nodal assessment can be technically challenging—especially for nodes within the preserved segments—preoperative identification of patients at high risk for VPI is clinically valuable. When VPI is suspected preoperatively, surgeons may adopt a more cautious approach to nodal evaluation or consider alternative surgical strategies.

(Line 55, page 5)

The incidence of lymph node metastasis is correlated with VPI (3). Its prognostic value is reflected in the T category of the TNM classification for lung cancer (4). In the era of increasing use of segmentectomy for small-sized NSCLC, accurate preoperative VPI identification is critical, as lymph node metastasis generally excludes eligibility for segmentectomy and intraoperative nodal assessment can be difficult, especially for nodes within or near the preserved segments. Accurate preoperative identification of VPI is essential for appropriate surgical decision-making and has a significant impact on treatment strategies (5 , 6).

**Comment 4:**

**The manuscript states that 3D AI imaging offers advantages over 2D but does not clearly explain what is gained by the 3D approach. Please clarify which specific features or metrics benefit from 3D analysis, and why they cannot be captured in 2D AI-based models.**

**Response 4:**

Thank you so much for your valuable comment. We have clarified in the Discussion that, in addition to calculating total and solid component volumes, the Vincent AI system also performs a comprehensive analysis of 22 imaging features derived from 3D CT data. To automatically generate descriptions of lung nodules, the system extracts detailed imaging findings from 3D reconstructions using three modules: (1) bronchopulmonary segment prediction AI, (2) nodule classification AI, and (3) finding description generation AI. This capability allows precise and objective quantification of morphological and contextual features that cannot be reliably obtained from 2D images. We have added this explanation to the manuscript.

(Line 370, page 34)

Our approach utilizes the advantages of 3D image analysis and overcomes the limitations of 2D-CT slice assessment. Unlike 2D analysis, which assesses only selected slices, 3D analysis evaluates the entire tumor volume in three dimensions, enabling accurate characterization of its overall morphology and spatial relationship to the pleura. Moreover, the AI software beta version of Synapse Vincent system calculates total and solid component volumes and extracts 22 imaging features from 3D reconstruction images using three modules: bronchopulmonary segment prediction, nodule classification, and finding description generation. These metrics cannot be reliably obtained from single-slice 2D-CT (26). For VPI prediction, it is essential to differentiate between a single area of pleural attachment and an extended area of attachment, because the latter has a greater risk of pleural invasion (33).

**Comment 5:**

**The distinction between “round shape” and “smooth shape” is unclear. Are these mutually exclusive? Are they referring to surface regularity versus contour circularity? Please define these features precisely, preferably with visual examples or definitions.**

**Comment 6:**

**The term “fatness” in Table 2 and results is unclear. Does this refer to fat-containing nodules or another imaging characteristic? Please clarify or revise this term for precision.**

**Response 5 and 6:**

Thank you for your thoughtful comments. As also noted in our response to Reviewer #2, we have revised the manuscript to include a detailed description of the 22 AI-derived radiological features used in the analysis. To enhance clarity and reproducibility, we have also added a supplement figure (Figure S5) illustrating representative examples of each feature. We have created a categorized list of the features and added it to the supplemental method.

(Supplementary Methods, Line 65, page 5)

This AI lung nodule analysis model uses a convolutional neural network based on VGG-16 and consists of 12 convolution layers, with four layers removed from the output side of the VGG-16. To extract 3D imaging features, 3D convolution was used for all convolution layers.

A detailed description and figure of the 22 AI-derived radiological features used in the analysis is provided below (Fig S5). These features were automatically extracted from CT images using the AI algorithm. Following the methodology described in reference (7), representative image examples with high AI confidence scores were selected for each of the 22 features.

**Irregular shape**: Tumors with an asymmetrical or uneven contour.

**Round shape**: Tumors with a generally circular or oval outline.

**Smooth shape**: Tumors with a uniformly flat and regular surface.

**Clear boundary**: The tumor surface is distinctly visible and can be clearly differentiated from the surrounding lung parenchyma.

**Serrated edge**: Tumor edge exhibits a saw-tooth-like appearance.

**Spiculation**: Thin lines extending from the edge of the tumor into the surrounding lung tissues.

**Lobulated edge**: Tumor border appears multilobulated.

**Polygon edge**: Tumor outline displays multiple straight segments forming polygonal shapes.

**Bronchus translucency**: Presence of bronchial air passages visible within the tumor.

**Bronchial convergence**: Bronchi appear to converge toward the tumor mass.

**Bronchial compression**: Narrowing of bronchi due to tumor pressure.

**Pleural indentation**: Local retraction of the pleura toward the tumor.

**Pleural contaction**: Tumor contact to the pleural surfaces.

**Pleural recess**: Tumor extension into the pleural recesses.

**Pleural hypertrophy**: Thickening of pleura adjacent to the tumor.

**Solid**: Tumor appears completely opaque without air or fluid components.

**Part-solid**: Tumor contains both solid and ground-glass components.

**Ground-glass opacity (GGO)**: Tumor area with hazy increased attenuation that does not obscure underlying structures.

**Cavity**: Air-filled space within the tumor mass.

**Calcification**: Presence of high-density foci within the tumor indicating calcified material.

**Fat component**: Tumor shows areas with fat-density attenuation.

(Supplementary Methods, Line 204, page 13)

**Fig S5. This is the CT images of representative examples of 22 AI-derived radiological features used in the analysis.** This is the fig S5 legend. This figure illustrates representative CT images demonstrating the 22 radiological features automatically extracted by the AI model. Each panel corresponds to one feature, with visual examples defining each characteristic.

**Comment 7:**

**Please include an explanation in the text for why “Pleural contact” and “Solid nodule” were chosen over others such as “spiculation” or “pleural indentation,” which are commonly associated with VPI.**

**Response 7:**

Thank you for your insightful comment regarding the selection of “pleural contact” and “solid nodule” as predictors over features such as “spiculation” or “pleural indentation.” In our study focusing on tumors adjacent to the pleura, “pleural contact” and “solid nodule” emerged as the main predictors in stability selection analysis. Although pleural indentation and spiculation are generally considered associated with VPI, they did not reliably indicate its presence in this specific setting. We believe that focusing on pleura-adjacent lesions better reflects the real-world clinical challenge of determining VPI when tumors are already close to the pleura. We have added this explanation to the manuscript.

(Line 405, page 36)

However, we excluded lesions not in close contact with the pleura to avoid an artificially high VPI detection accuracy, reflecting the true complexity of identifying VPI in ambiguous clinical scenarios and reinforcing the applicability of our findings in real clinical situations. In tumors adjacent to the pleura, the primary predictors were identified as "pleural contact" and "solid nodule." While pleural indentation and spiculation are typically regarded as indicators of VPI, they did not consistently demonstrate its presence in this setting. Our approach of using AI for 3D image analysis to predict VPI is a robust platform for future developments in NSCLC diagnostics.

**Reviewer #4:**

**Comment 1: The authors present an interesting application of AI-driven 3D CT analysis for predicting visceral pleural invasion (VPI) in early-stage NSCLC. The authors should address the overfitting concerns through more rigorous validation and contextualize their work within the broader landscape of AI applications in lung cancer imaging.**

**The decrease in AUC from training (0.816, 0.831) to test (0.782, 0.767) cohort, combined with the drop in the key 'Solid nodule' feature performance (0.786→0.741), suggests potential overfitting that could limit clinical generalizability. Common solutions are 1) implement cross-validation within the training set to better estimate true model performance and detect overfitting earlier. 2) consider ensemble methods or additional regularization techniques to improve model stability, 3)report confidence intervals for all performance metrics and test for statistical significance of performance differences. Among these approaches, implementing cross-validation (option 1) is particularly essential and would substantially strengthen the validation framework.**

**Response 1:**

Thank you for your comment.

As you pointed out, a decrease in AUC was observed.

1)Cross-validation is indeed a widely used and effective method for evaluating the internal validity of a model. However, in this study, we intentionally did not use cross-validation for model evaluation.

Instead, the test cohort was defined based on the date of surgery, creating a temporal split. Unlike random partitioning as typically used in cross-validation, which assesses internal validity alone, our approach allows for an additional evaluation of external validity. As such, we believe this provides a more reliable measure of model performance.

This approach is referred to as "temporal validation", and is described in the TRIPOD statement as a method that lies between internal and external validation.

Considering that the final model is relatively simple, we believe that the observed decrease in AUC is more likely due to a shift in patient distribution over time rather than overfitting. The decrease in AUC for the "Solid nodule" feature likely reflects the same cause.

Importantly, despite the shift in patient characteristics, the change in AUC was less than 0.05, which we believe supports not only the internal validity but also the external validity of the proposed model.

2) We appreciate your suggestion regarding ensemble methods and enhanced regularization.

Regularization is already incorporated into our modeling through the use of Lasso regression within the stability selection framework. By combining repeated subsampling with variable selection, this method provides more stable identification of variables that are likely to be truly associated with VPI, compared to applying Lasso only once.

While ensemble methods may potentially improve predictive accuracy, they often lead to increased model complexity and decreased interpretability, especially in terms of variable contributions. Given our goal of producing a clinically interpretable model, we did not pursue ensemble learning.

3) Regarding model performance, we have already reported confidence intervals. Given the substantial overlap of these intervals across models, we do not consider additional statistical testing to be necessary.

**Comment 2:**

**The manuscript would also benefit from broader contextualization within the growing field of deep learning applications for lung cancer CT imaging. Given that both studies employ deep learning on CT imaging for lung cancer applications, citing 'Improving lung cancer diagnosis and survival prediction with deep learning and CT imaging' would provide valuable context for how AI-driven approaches are advancing across different aspects of lung cancer management, from diagnosis to prognosis to surgical planning.**

**Response 2:**

Thank you so much for the helpful comment. As the reviewer suggested, we have added the recommended article for citation as follows.

(Line 74, page 6)

Recently, AI has significantly contributed to advancements in the medical field (17), assisting in highly precise evaluation of radiological diagnoses and providing objective radiological findings (19,20).

**General Editorial Comments**

**Comment 3:**

**“Thank you for stating the following financial disclosure:**

**“Norihiko Ikeda reports receiving a research grant from the FUJIFILM Corporation for the Department of Surgery, Tokyo Medical University.”**

**Please state what role the funders took in the study. If the funders had no role, please state: "The funders had no role in study design, data collection and analysis, decision to publish, or preparation of the manuscript."**

**Response 3:**

Thank you for the comment. The funders had no role in the study, so we will add the following phrase:

The funders had no role in study design, data collection and analysis, decision to publish, or preparation of the manuscript.

**Comment 4:**

**“Thank you for stating the following in the Competing Interests section:**

**“Norihiko Ikeda reports receiving a research grant from the FUJIFILM Corporation for the Department of Surgery, Tokyo Medical University. Although we have utilized the AI software integrated within the Synapse Vincent System (Fujifilm Corporation, Japan) for this study, it is essential to note that this is a development model and not a commercially available product. We have no commercial conflicts of interest to disclose.”**

**Please confirm that this does not alter your adherence to all PLOS ONE policies on sharing data and materials, by including the following statement: “This does not alter our adherence to PLOS ONE policies on sharing data and materials.” (as detailed online in our guide for authors** [**http://journals**](http://journals)**.plos.org/plosone/s/competing-interests).”**

**Response 4:**

Thank you for the comment. As recommended, we will include the following phrase:

This does not alter our adherence to PLOS ONE policies on sharing data and materials.

**Comment 5:**

**“In the online submission form you indicate that your data is not available for proprietary reasons and have provided a contact point for accessing this data. Please note that your current contact point is a co-author on this manuscript. According to our Data Policy, the contact point must not be an author on the manuscript and must be an institutional contact, ideally not an individual. Please revise your data statement to a non-author institutional point of contact, such as a data access or ethics committee, and send this to us via return email. Please also include contact information for the third party organization, and please include the full citation of where the data can be found.”**

**Response 5:**

Thank you for the comment. As recommended, we will include the following phrase:

The data underlying this article cannot be shared publicly due to ethical restrictions under the policies of Tokyo Medical University. Data are available from the Ethics Committee of Tokyo Medical University for researchers who meet the criteria for access to confidential data.

**Comment 6:**

**“Please upload a copy of Figure 3 and 4, to which you refer in your text on page 25 and 28. If the figure is no longer to be included as part of the submission please remove all reference to it within the text.”**

**Response 6:**

We thank the reviewer for this comment and apologize for the error in figure labeling. The figures referred to as Figures 3 and 4 in the original manuscript were incorrectly labeled as Figures 2A and 2B. We have corrected the figure labels and updated all corresponding references in the text.
